# MT1-MMP is the critical determinant of matrix degradation and invasion by ovarian cancer cells

**DOI:** 10.1038/sj.bjc.6603863

**Published:** 2007-07-03

**Authors:** K L Sodek, M J Ringuette, T J Brown

**Affiliations:** 1 Department of Cell and Systems Biology, University of Toronto, 25 Harbord Street, Toronto, Ontario, Canada M5S 3G5; 2 Department of Obstetrics and Gynecology, Samuel Lunenfeld Research Institute, 600 University Avenue, Suite 876, Toronto, Ontario, Canada M5G 1X5

**Keywords:** MT1-MMP, ovarian cancer, invasion, motility, collagen I, E-cadherin

## Abstract

Membrane-type 1 matrix metalloproteinase (MT1-MMP), a transmembrane metalloprotease that plays an important role in the invasion of many solid tumour types, promotes pericellular matrix degradation and may also stimulate tumour cell motility. As both these processes are key contributors to intraperitoneal ovarian tumour metastasis, we examined six ovarian cancer cell lines to determine whether MT1 is a critical mediator of invasive behaviour for this tumour type. Our results indicated that only those cell lines that expressed MT1 were capable of penetrating a type I collagen barrier, with the capacity for both matrix degradation and invasion reflecting endogenous MT1 expression level. Ectopic MT1 expression endowed an invasive phenotype upon cell lines lacking MT1 that were previously non-invasive, indicating the crucial role of this protease. Conversely, invasion was abolished by tissue inhibitor of metalloproteinase-2 (TIMP-2), a potent inhibitor of MT1, yet was minimally affected when other (secreted) MMPs were inhibited using TIMP-1 and the gelatinase inhibitor SB-3CT. Whereas collagen I degradation was strikingly accelerated by ectopic MT1 expression, cell motility remained unchanged. We conclude that MT1 is necessary for collagen I invasion by ovarian cancer cells, and that its requisite activity is the promotion of matrix degradation, with no impact on cell motility.

Epithelial ovarian cancer is a highly aggressive malignancy associated with a poor prognosis. These tumours have usually metastasised before clinical presentation, and fewer than 30% of patients diagnosed with metastatic disease survive beyond 5 years (Landis *et al*, 1999), as present therapeutic strategies are ultimately ineffective in preventing disease progression. In contrast to most other solid tumours, which metastasise through a multi-step haematological route, ovarian cancer cells metastasise via exfoliation from the primary tumour into the peritoneal cavity. Ovarian cancer cells preferentially bind collagen I and this ligand is highly effective in stimulating motility in these cells (Moser *et al*, 1996; Burleson *et al*, 2004). Indeed, during peritoneal metastasis, tumour cells attach preferentially at locations where the mesothelium is disrupted and the underlying collagen I-rich stromal matrix is exposed (Ghosh *et al*, 2002; Mochizuki *et al*, 2004).

Metastatic cancer cells have acquired the proteolytic mechanisms required for penetration of basement membrane and collagen I-rich stromal matrices, which are normally refractory to invasion by epithelial cells (Sivridis *et al*, 2004). Matrix metalloproteases (MMPs), key mediators of ECM degradation (Egeblad and Werb, 2002), are upregulated in invasive cancers including those of ovarian origin (Ghosh *et al*, 2002). Although the importance of MMPs in ovarian cancer metastasis is recognised (Stack *et al*, 1998; Ghosh *et al*, 2002), clinical trials that have evaluated the broad-range MMP inhibitors marimastat and tanomastat for cancer treatment, including ovarian, have yielded disappointing results (Coussens *et al*, 2002; Hirte *et al*, 2006). This may be partially attributed to the concomitant inhibition of critical physiological processes involved in combating tumour progression (Overall and Lopez-Otin, 2002). It is therefore necessary to identify which subsets of MMPs play a critical role in ovarian cancer metastasis, so that they can be specifically targeted. A central role for a transmembrane member of the MMP family, membrane-type 1 MMP (MT1), in cancer metastasis has emerged in association with the aggressive behaviour of several cancer types (Aznavoorian *et al*, 2001; Seiki, 2003; Ueda *et al*, 2003; Sabeh *et al*, 2004; Cao *et al*, 2005), as reviewed by Sounni and Noel (2005). We therefore sought to determine whether this protease plays an essential role in regulating ovarian cancer cell invasive behaviour.

The potency of MT1 owes to the ability of its proteolytic activity to be focused, via its translocation to the leading edge of invasive cancer cells (Mori *et al*, 2002). Indeed, MT1 is an essential functional component of invadopodia (Chen and Wang, 1999; Artym *et al*, 2006). Although MT1 is known for its potent collagenolytic activity, it cleaves a range of additional ECM substrates and cell surface-associated receptors. Membrane-type 1 MMP also activates soluble MMPs including pro-MMP-2, a gelatinase that cooperates with MT1, to degrade stromal and basement membrane barriers (Ohuchi *et al*, 1997; Seiki, 2003). The resulting proteolysed matrix, in turn, promotes further tumour growth and invasion (Hornebeck and Maquart, 2003).

Although matrix degradation is an obligatory aspect of tumour cell invasion, cell motility is an additional important contributor. Membrane-type 1 MMP has been implicated in enhancing cell motility through cleavage of cell surface receptors, such as CD44 and syndecan-1, and through generation of haptotactic signals by cleavage of laminin and collagen IV (Gingras *et al*, 2001; Rozanov *et al*, 2001; Ueda *et al*, 2003; Takino *et al*, 2004). Consistent with these observations, MT1 overexpression in some cancer cell types appears to promote motility on collagen I and fibronectin matrices, although the requirement for its enzymatic activity in this process is controversial (Giannelli and Antonaci, 2000; Kajita *et al*, 2001; Endo *et al*, 2003; Hornebeck and Maquart, 2003; Koshikawa *et al*, 2005; cf. Cao *et al*, 2004). Collectively, these studies suggest that MT1 may coordinate two cell activities necessary for invasion – matrix degradation and cell motility.

Although the functionality of MT1 in many solid tissue cancers is well established, information on its role in ovarian cancer is limited. Membrane-type 1 expression by cells of orthotopically implanted human ovarian cancer biopsies has been linked to a more aggressive tumorigenicity (Drew *et al*, 2004), and inhibition of MT1 activity has been shown to block collagen invasion in one ovarian cancer cell line, DOV-13 (Ellerbroek *et al*, 1999; Ellerbroek *et al*, 2001). However, as different ovarian cancer cell lines display varying capacities for peritoneal tumour formation *in vivo* (Shaw *et al*, 2004), and may use different mechanisms for invasion, we sought to determine whether MT1 expression determines invasive behaviour within a diverse panel of human ovarian cancer cell lines. A polymerised collagen I matrix was used to assess invasion for several reasons: Collagen I plays a key involvement in ovarian cancer metastasis, being the most abundant matrix molecule in the submesothelial stroma, and is the preferred substrate for ovarian cancer cell attachment during peritoneal metastasis (Moser *et al*, 1996). Furthermore, the common use of Matrigel for invasion assays has been brought into question as it does not form a protease-dependent barrier and therefore does not adequately represent a basement membrane (Hotary *et al*, 2006).

Although motility is an important aspect of ovarian cancer peritoneal dissemination, the potential promotion of motility by MT1 in ovarian cancer cells has not been assessed. Therefore, the impact of MT1 expression on motility, in addition to collagen I degradation, was evaluated. As elevated E-cadherin is known to suppress invasive behaviour of cancer cells and to negatively regulate MMP expression, including that of MT1 (Ara *et al*, 2000; Takahashi *et al*, 2002; Nawrocki-Raby *et al*, 2003a, 2003b; Hazan *et al*, 2004; Hlubek *et al*, 2004; Munshi and Stack, 2006), we also assessed cadherin expression in these cell lines.

Our results demonstrate that MT1 activity is the prime determinant of ovarian cancer cell invasion through a collagen I matrix, and that this effect occurs through enhancement of matrix degradation without impacting cell motility.

## MATERIALS AND METHODS

### Cell culture

Human ovarian cancer cell lines HEY, SKOV-3 and HOC-7 (obtained from Dr Alexander Marks, University of Toronto, Toronto, ON, Canada), OVCA429 (from Dr Robert Kerbel, Sunnybrook Hospital, Toronto, ON, Canada), OVCAR-3 and ES-2 (from American Type Culture Collection, Manassas, VA, USA) were maintained in *α*-minimal essential media (*α*-MEM; GIBCO, Invitrogen Corp., Mississauga, ON, Canada) supplemented with 10% foetal bovine serum (FBS; Cansera International Inc., Etobicoke, ON, Canada), 0.017% penicillin G and 0.01% gentamycin at 37°C and 5% CO_2_.

### RT–PCR

RNA was extracted from confluent cells grown on collagen I films using an RNeasy mini kit (Qiagen, Mississauga, ON, Canada) and reverse transcribed using a First Strand cDNA Synthesis Kit (Invitrogen Corp.). Primers for MT1 were: forward 5′-atcaacactgcctacgagag-3′ and reverse 5′-aagacttcatcgctgcccat-3′ (310 bp amplicon). Primers for E-cadherin were: forward 5′-tccatttcttggtctacgcc-3′ and reverse 5′-caccttcagccaacctgttt-3′ (361 bp amplicon). Primers for RPL13a, used as an endogenous control gene (Mogal A, 2006) were: forward 5′-catcgtggctaaacaggtactg-3′ and reverse 5′-gcacgaccttgagggc-agcc-3′ (319 bp amplicon). An annealing temperature of 59^o^C was used for all primers. Samples were taken at various cycles to ensure comparisons were made during the log phase of amplification. For MT1, 26–30 cycles were used; for RPL13a, 26 cycles and for E-cadherin 32 cycles. Real-time PCR for MT1 was performed using the TaqMan® Gene Expression Assay system (Applied Biosystems, Foster City, CA, USA) according to the manufacturer's instructions, using validated probes human MT1 (no. 4331182) and eucaryotic 18S endogenous control (no. 4319413E).

### Western blotting

Confluent cells were lysed in 50 mM Tris-HCl, 120 mM NaCl, 0.5% NP-40, pH 7.4, containing protease inhibitor cocktail (Sigma-Aldrich, St. Louis, MO, USA). Total protein was quantified by the Bio-Rad Protein Assay (Bio-Rad Laboratories, Mississauga, ON, USA). Equal amounts of protein (10–20 *μ*g) were separated by 12% SDS–PAGE and transferred onto PVDF membranes (Amersham Biosciences, Oakville, ON, Canada), which were probed for MT1 (1 : 1000, Ab815; Chemicon International Inc., Temecula, CA, USA), E-cadherin (1 : 1000, Chemicon), N-cadherin (1 : 2500, Signal Transduction Laboratories, Lexington, KY, USA) or *β*-tubulin (1 : 2000, T5168, Sigma-Aldrich). Secondary HRP-coupled antibodies (Amersham, Oakville, ON, Canada) were diluted 1 : 3000. Immunoreactive proteins were visualised using ECL Western Blotting Detection Reagents (Amersham Biosciences).

### Gelatin zymography

Capacity for pro-MMP-2 activation was evaluated as a means of assessing MT1 activity. This assay was performed using cells seeded on 3D collagen I gels as is necessary for stimulation of this event (Ellerbroek *et al*, 1999; Ellerbroek *et al*, 2001; Zigrino *et al*, 2001). Vitrogen™ (eight volumes of 3 mgml^−1^) was diluted with 1 volume of 10 × *α*MEM and one volume of 0.1 M NaOH, and the neutralised collagen I solution was polymerised at 37°C for 1 h (100 *μ*l well^−1^; 48-well plate). Cells (1 × 10^5^) were seeded atop the collagen I gels in 300 *μ*l media that had been pre-conditioned for 24 h by human gingival fibroblasts (HGF), in order to provide an exogenous source of pro-MMP-2 and tissue inhibitor of metalloproteinase-2 (TIMP-2). Additional aliquots of cells were seeded into wells for later quantification, to verify equal cell numbers had been present during the assay, allowing a meaningful comparison of pro-MMP-2 activation in the conditioned media between the ovarian cancer cell lines. Following a 36-h incubation, media was collected, diluted in non-reducing sample buffer, and 20 *μ*l aliquots were separated by 12% SDS–PAGE using gels co-polymerised with 0.016% gelatin (Sigma-Aldrich). Gels were washed twice for 10 min in 2.5% Triton X-100, rinsed in dH_2_O, and incubated in enzyme activation buffer (50 mM Tris-HCl, pH 7.4, containing 0.2 M NaCl, 5 mM CaCl_2_, and 0.166% Brij 35) for 24–48 h at room temperature. Coomassie blue (0.5%) staining revealed proteins with gelatinolytic activity (gelatin clearance).

### Transwell collagen invasion

Experiments were conducted in 8 *μ*m transwell chambers (Costar, Corning Inc., Corning, NY, USA). Vitrogen™ (Cohesion, Palo Alto, CA, USA) was diluted in ice-cold PBS and neutralised with 0.01 N NaOH for a final concentration of 0.2 *μ*g *μ*l^−1^, of which 100 *μ*l was applied to each transwell membrane, polymerised by incubation at 37°C for 1 h, and dried to form a compact collagen I film on the membrane. The coated membranes were rinsed with PBS and equilibrated in serum-free *α*-MEM using pre-warmed solutions. Cells were resuspended in *α*-MEM containing 1% FBS, and 1 × 10^5^ cells in 100 *μ*l were seeded into the upper wells. *α*-MEM (500 *μ*l) supplemented with 10% FBS was used as a chemoattractant in the lower well. Where applicable, the MMP inhibitor GM6001 (Chemicon) was added at 25 *μ*M, and TIMP-1 and TIMP-2 (obtained from Dr Chris Overall, University of British Colombia, Vancouver, BC, Canada) were used at 2.5 *μ*g/ml. SB-3CT (MMP2/MMP-9 inhibitor IV, Chemicon), which selectively inhibits the gelatinases MMP-2 and MMP-9 (Kleifeld *et al*, 2001) was used at 6 *μ*M. At the time of harvest (24–72 h), invaded cells were released from the bottom of the transwell insert using 500 *μ*l of 0.08% trypsin (in a 24-well plate), collected by centrifugation, and invaded cells were quantified based on nucleic acid content using CyQUANT™ (Molecular Probes Inc. Eugene, OR, USA) according to the manufacturer's instructions. Invasion experiments were performed three times, with data normalised to HEY cell invasion levels and combined for the three experiments.

### Collagen I degradation

Chamber slides were coated with Vitrogen™ that had been neutralised with 0.01 M NaOH and diluted in PBS such that 200 *μ*l containing 20 *μ*g collagen I were added to each well. The collagen was subsequently polymerised at 37°C for 1 h and dried down overnight to create compact collagen I films. Transwells were coated with collagen as described above for transwell invasion. The collagen matrices were biotinylated under sterile conditions, using 20 *μ*g/ml EZ-Link Sulfo-NHS-LC-LC-Biotin (Pierce, Rockford, IL, USA) in 50 mM sodium bicarbonate pH8.3 for 2 h, quenched using 50 mM Tris, pH7.5, and washed before addition of cells. For examination of pericellular collagen degradation, cells were seeded into chamber slide wells at low density (1 × 10^3^ cells well^−1^) in *α*-MEM containing 5% FBS. To assess collagen removal from transwell pores, cells were seeded in transwell chambers as described above for transwell invasion. Following incubation for 18–72 h, matrix degradation experiments were terminated by fixation in 4% paraformaldehyde, permeabilised with 0.1% Triton X-100/PBS, blocked in 2% BSA/PBS, and stained with streptavidin-Alexafluor-488 and rhodamine-phalloidin (Molecular Probes Inc.), each diluted 1 : 200 in 2% BSA/PBS, then visualised by confocal laser scanning microscopy (LSM 510, Carl Zeiss Inc., Toronto, ON, Canada).

### Ectopic MT1 expression

Membrane-type 1 expression constructs encoding wild-type flag-tagged MT1 (MT1f) and catalytically inactive MT1 (MT1f-E240A) (Tam *et al*, 2002), generated by the lab of Dr M Sharon Stack (Wu *et al*, 2004), were obtained from Dr C Overall. Cells were transfected using Fugene-HD (Roche Applied Science, Laval, PQ, Canada) according to the manufacturer's instructions. Additional control cells were transfected with pEGFP (Clontech, Mountain View, CA, USA) or left untransfected. Cells were seeded for migration, collagen degradation, or invasion assays 24–36 h post-transfection.

### Cell migration

Cell migration on collagen was assessed using transwell membranes coated with unpolymerised collagen I (Vitrogen™) as described for the invasion assay, except the collagen I was neither neutralised nor incubated at 37°C before being dried onto the membrane. Cell seeding, application of chemoattractant, and quantification of migrated cells were performed as described for the transwell invasion assay, and experiments were terminated at 8–10 h. As with the invasion assays, the migration assays were initiated 24–48 h post-transfection (where applicable) to allow for cell recovery and ectopic expression of MT1. Migration experiments were repeated three times.

## RESULTS

### Characterisation of MT1 expression and activity

**Figure 1:**
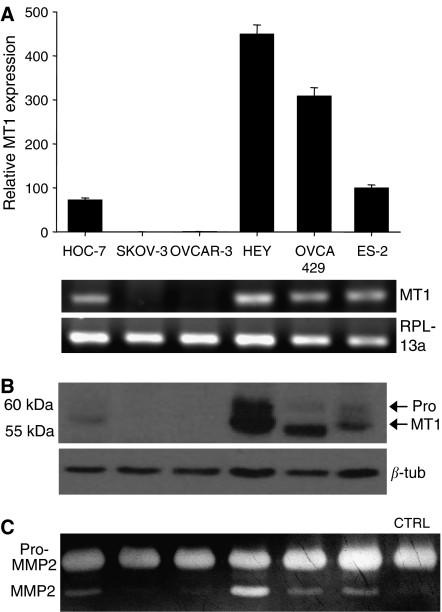
Membrane-type 1-MMP expression in ovarian cancer cell lines. (**A**) Real-time PCR results for MT1 expression by ovarian cancer cell lines grown on a collagen I film. Results of RT–PCR for MT1 are depicted below, with amplification of the endogenous control gene RPL13a shown to confirm the use of equal template in all reactions. (**B**) Western blot with polyclonal antibody Ab815 showing detection of MT1 expression in lysates of cells grown on a collagen I film. The blot was re-probed for *β*-tubulin to verify equal loading. (**C**) Gelatin zymography for pro-MMP-2 activation by cells grown on a 3D collagen I gel and incubated with HGF-conditioned media as an exogenous source of pro-MMP-2. Levels of pro-MMP-2 provided in the HGF-conditioned media are shown in the CTRL lane (in absence of cells).

Four of the six ovarian cancer cell lines examined, HEY, OVCA429, ES-2, and HOC-7, expressed MT1 as determined both by real-time PCR (Figure 1A) and Western blotting (Figure 1B). HEY cells expressed the highest levels of MT1, whereas expression was not detected in either the SKOV-3 or OVCAR-3 cells. To further confirm the expression of MT1, a well-established assay that is based on the capacity of MT1 to cleave and activate pro-MMP-2 was performed (Ellerbroek *et al*, 1999; Ellerbroek *et al*, 2001; Zigrino *et al*, 2001). The activation of pro-MMP-2 involves binding of a TIMP-2/pro-MMP-2 dimer to MT1, presenting the pro-MMP-2 to a second MT1 molecule, which cleaves the pro-domain from MMP-2 (Strongin *et al*, 1995). The pro-MMP-2 activation results paralleled the MT1 expression pattern; only the cell lines that expressed MT1 (HEY, OVCA429, ES-2, and HOC-7) were able to cleave pro-MMP-2, with HEY demonstrating the most extensive activity (Figure 1C).

### Transwell collagen I invasion and matrix degradation correlate with MT1 expression

**Figure 2:**
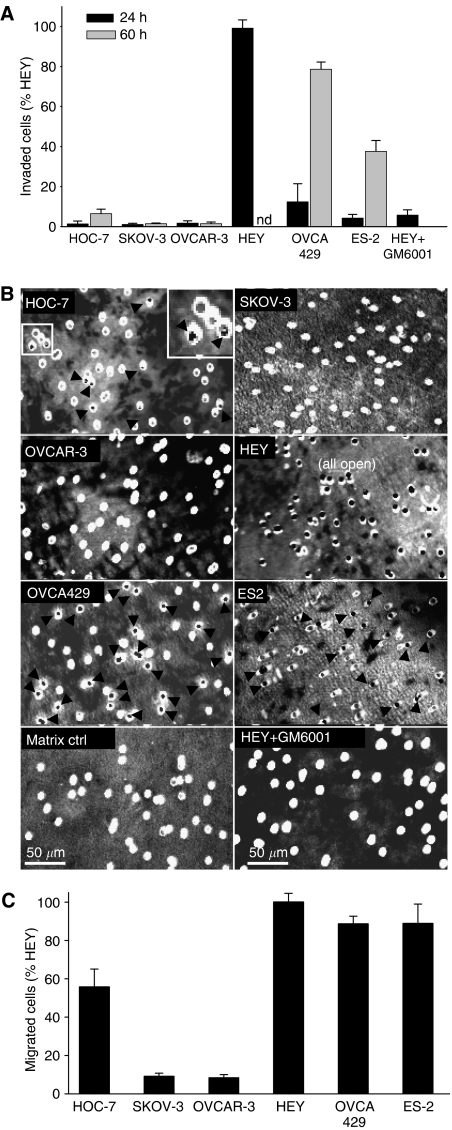
Collagen I transwell invasion, matrix degradation and motility of ovarian cancer cell lines. (**A**) Collagen I transwell invasion capacity. Results from three independent experiments were normalised to HEY and data were pooled. Bars represent the mean±s.e. (nd – HEY invasion was not assessed beyond 24 h as invasion was complete by this time). (**B**) Clearance/degradation of collagen I plugs from transwell invasion membranes. Confocal fluorescence microscopy was used (× 16) to visualise the biotinylated collagen. Representative areas near the centre of the membrane are shown and arrows highlight transwell pores that have been cleared of their collagen I plugs, except for HEY, in which all pores were cleared. Inset shows an enlarged image that distinguishes cleared pores (black) from uncleared pores (grey). (**C**) Transwell migration through unpolymerised collagen I-coated transwells at 8–10 h. Results from three independent experiments were normalised to HEY and data were pooled. Bars represent the mean±s.e.

The relationship between MT1 expression and invasive capacity of ovarian cancer cell lines was assessed using a transwell collagen invasion assay. HEY cells had extensively penetrated the collagen I barrier by 24 h (Figure 2A). In comparison, OVCA429 and ES-2 cells required twice as long to accomplish moderate levels of invasion, whereas HOC-7 cells rarely invaded. SKOV-3 and OVCAR-3 cells, which do not express MT1, did not invade, even when incubated for up to 5 days (data not shown). Invasion was abolished (>95%) by the presence of GM6001 in the culture medium (Figure 2A), with similar effects obtained when OVCA429 and ES-2 cells were used (data not shown). These studies demonstrate an essential role of MMP-mediated proteolysis for invasion of these collagen I matrices.

To examine the relationship between transwell collagen invasion and matrix degradation, the ability of cells to clear the collagen plugs within pores of collagen-coated transwell membranes was assessed. Only those cell lines expressing MT1 removed collagen from the transwell pores (Figure 2B), consistent with the transwell invasion results. HEY cells showed extensive clearing of collagen from the pores by 24 h, whereas OVCA429, ES-2, and HOC-7 cells required a longer incubation to accomplish collagen degradation. By 55 h, HEY cells had cleared essentially all pores, OVCA429 cells had cleared 40–50% of pores and ES-2 and HOC-7 cells had cleared only 10–20% of pores. In contrast, SKOV-3 and OVCAR3 cells were unable to clear the pores. Incubation of HEY cells with GM6001 prevented collagen clearing (Figure 2B), with similar results obtained for OVCA429 and ES-2 cells.

### Cell lines expressing endogenous MT1 generally show higher motility on collagen I

As MT1 promotes the motility of several cancer cell types on various matrices including collagen (Gingras *et al*, 2001; Rozanov *et al*, 2001; Takino *et al*, 2004), we evaluated this potential association within our panel of ovarian cancer cell lines. For these experiments, we assessed the ability of the cells to transverse transwell membranes coated with collagen I that was left unpolymerised, to eliminate the prerequisite of proteolysis. SKOV-3 and OVCAR-3 cells were significantly less motile than the MT1-expressing cell lines. However, within the MT1-expressing cell lines, there was not a clear correlation between the level of MT1 expression and motility. HEY cells, which express the highest levels of MT1, exhibited the same level of motility as OVCA429 and ES-2 cells, which express much lower levels of MT1 (Figure 2C).

### Inhibition of MT1 activity prevents invasion

**Figure 3:**
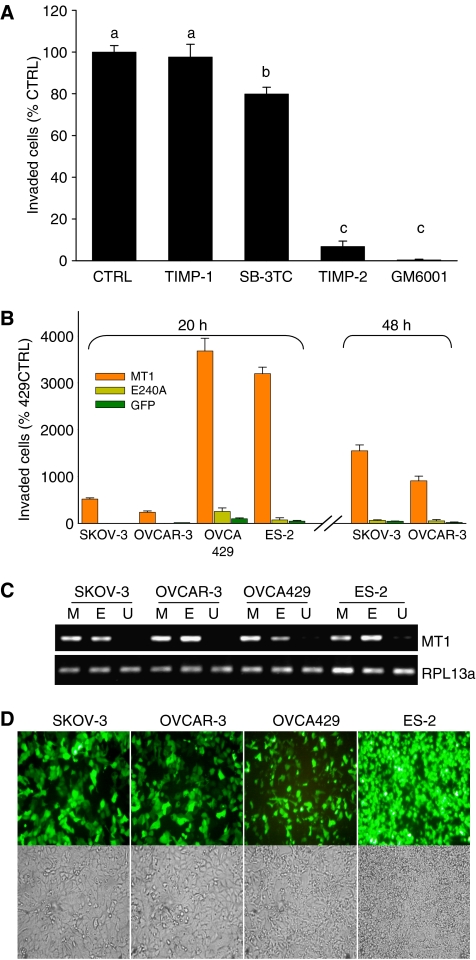
A critical role for MT1 in collagen I invasion by ovarian cancer cells. (**A**) Selective inhibition of MT1 blocks invasion. HEY cells were incubated with the indicated inhibitors during a 24 h invasion assay. Data were analysed by one-way ANOVA followed by Student–Newman–Keuls multiple comparison test. Bars with different letters are statistically different from one another. (**B**) Expression of catalytically active MT1 is sufficient to cause invasion by the non-invasive SKOV-3 and OVCAR-3 cells, and also markedly enhances invasion by the moderately invasive OVCA429 and ES-2 cells. In contrast, expression of the MT1 catalytically inactive mutant, E240A, had no effect on invasion compared with the GFP-transfected control. (**C**) Verification of MT1 and E240A expression by transfected cells. RT–PCR for MT1 shows comparable expression of the MT1f (M) and the E240A mutant (E) were achieved, both of which were expressed far in excess of endogenous (U) MT1 levels of OVCA429 and ES2. (**D**) The transfection efficiency exceeded 50% for all cell lines, shown using fluorescence microscopy of the GFP-transfected control cells. The upper panel shows GFP fluorescence indicating transfected cells, whereas the lower panel shows all cells in the field by light microscopy.

The contribution of MT1 to invasion was assessed using a variety of MMP inhibitors. HEY cells were chosen for these experiments, as they consistently exhibited the most invasive behaviour. Invasion was abolished by the broad-range MMP inhibitor GM6001, indicating the essential role of MMP-mediated proteolysis in this process (Figure 3A). Tissue inhibitor of metalloproteinase-1, which does not affect MT1 activity (Will *et al*, 1996), but is an effective inhibitor of all secreted MMPs (Hornebeck *et al*, 2005), did not affect invasion. Although the selective inhibition of the gelatinases, MMP-2 and MMP-9 with SB-3CT (Kleifeld *et al*, 2001) resulted in a 20% reduction in invasion, this decrease was modest in comparison to the reduction attained with TIMP-2, which prevented invasion as effectively as GM6001 (>90% inhibition). In contrast to SB-3CT and TIMP-1, GM6001 and TIMP-2 are potent inhibitors of MT1 (Will *et al*, 1996). Collectively, these results indicate a critical requirement of MT1, and not other MMPs, for collagen I invasion.

### Membrane-type 1 expression activates invasive behaviour by non-invasive cells

As our experiments indicated that inhibition of MT1 activity prevented invasive behaviour, we next examined whether MT1 was a limiting factor for ovarian cancer cell invasion. Both the non-invasive SKOV-3 and OVCAR-3 cells and the moderately invasive OVCA429 and ES-2 cell lines were transfected with either wild-type MT1 (MT1f) or catalytically inactive MT1 (E24A) expression constructs, and subjected to a transwell invasion assay. Comparable and high levels of expression were achieved with both constructs, at levels vastly exceeding endogenous MT1 expression by the invasive OVCA429 and ES-2 cell lines (Figure 3C). Transfection efficiency exceeded 50% for each of the four cell lines transfected, as shown using EGFP expression (Figure 3D). Membrane-type 1 overexpression by the moderately invasive OVCA429 and ES-2 cells caused a marked acceleration in their invasion. Moreover, ectopic expression of MT1 in the non-invasive SKOV-3 and OVCAR-3 cell lines activated an invasive phenotype (Figure 3B). Although MT1 overexpression allowed all cell lines to penetrate the collagen barrier within 24 h, the extent of invasion was correlated with motility. As SKOV-3 and OVCAR-3 cells were less motile, a longer incubation (48 h) was required to achieve extensive invasion by the MT1-transfected cells (Figure 3B). In contrast to MT1, overexpression of the E240A mutant was without effect, indicating the requirement for MT1 catalytic activity in the enhanced invasion.

### Membrane-type 1 promotes invasion solely through enhanced matrix degradation and not cell motility

**Figure 4:**
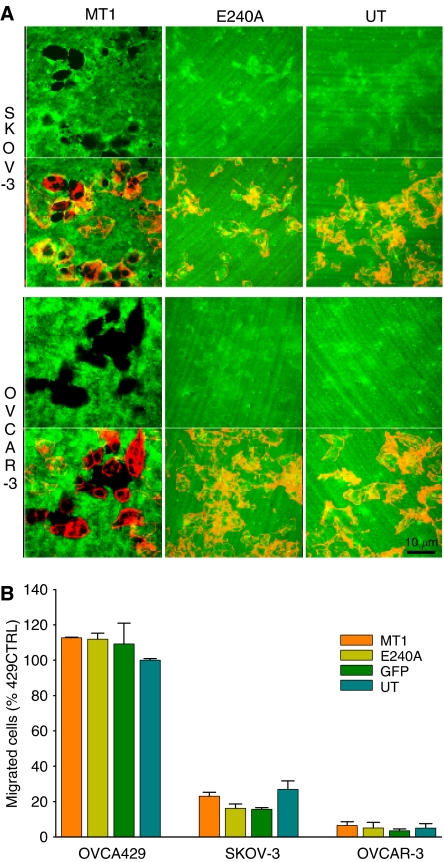
MT1 enhances invasion solely through its promotion of matrix degradation; not by affecting cell motility. (**A**) Degradation of biotinylated collagen I matrices by MT1-transfected SKOV-3 and OVCAR-3 cells after a 24 h incubation. Matrices were not degraded by cells expressing the E240A catalytically inactive MT1 mutant, nor by control untransfected (UT) cells. Cells were visualised via rhodamine phalloidin staining of actin (red) and biotinylated collagen matrices were visualised using streptavidin AF488 (green), using confocal microscopy (× 40). (**B**) Overexpression of MT1 or E240A did not affect cell motility on collagen. Cells were seeded in transwell chambers pre-coated with 20 *μ*g unpolymerised collagen and allowed to migrate for 8–10 h. Results shown are the mean±s.e. for three independent experiments.

Cell invasion can be partitioned into matrix degradation and cell motility. As MT1 has been implicated in both, we examined the impact of its overexpression on these processes independently to determine the mechanism through which MT1 promoted invasion. SKOV-3 and OVCAR-3 cells were transfected as described in the previous experiment. To assess matrix degradation, cells were seeded in chamber slides pre-coated with polymerised collagen that was biotinylated to allow visualisation. After 24 h, cells and matrices were fixed and analysed by confocal microscopy. Overexpression of MT1 resulted in a striking enhancement of collagen degradation, as shown by the cleared areas in Figure 4A. In comparison, non-transfected cells, and cells transfected with E240A, did not exhibit collagen clearance. Similar results were obtained when OVCA429 and ES-2 cells were transfected with these constructs (data not shown).

To determine whether MT1 also promoted the migration of ovarian cancer cells, as demonstrated in other cancer cell types (Gingras *et al*, 2001; Rozanov *et al*, 2001; Cao *et al*, 2004; Takino *et al*, 2004), the transfected cells were subjected to a transwell collagen migration assay. In contrast to its striking effect on collagen degradation, MT1 expression did not enhance cell migration (Figure 4B). Consistent with these observations, treatment of the MT1-expressing cells (HEY, 429, and ES-2) with GM6001 did not alter their motility (data not shown). Together, these data demonstrate that ovarian cancer cell motility is not promoted by MT1 proteolytic activity or expression.

### Invasive capacity does not relate to E-cadherin expression

E-cadherin is considered to be a tumour suppressor, as its expression is associated with increased cell–cell adhesion and decreased motility. The inability of SKOV-3 and OVCAR-3 cells to invade 3-D collagen gels may relate to their E-cadherin expression (Kokenyesi *et al*, 2003). That MT1 expression is upregulated through *β*-catenin signalling may explain the reported inverse correlation between E-cadherin and MT1 expression (Ara *et al*, 2000; Takahashi *et al*, 2002; Nawrocki-Raby *et al*, 2003a, 2003b; Hazan *et al*, 2004; Hlubek *et al*, 2004; Munshi and Stack, 2006). Therefore, we analysed cadherin expression to determine whether E-cadherin was associated with the absence of MT1 and cell invasion in SKOV-3 and OVCAR-3 cells. The expression of N-cadherin was also assessed, as this cadherin sub-type has been associated with motile carcinoma cells that have undergone epithelial-mesenchymal transition (Derycke and Bracke, 2004; Hazan *et al*, 2004).

**Figure 5:**
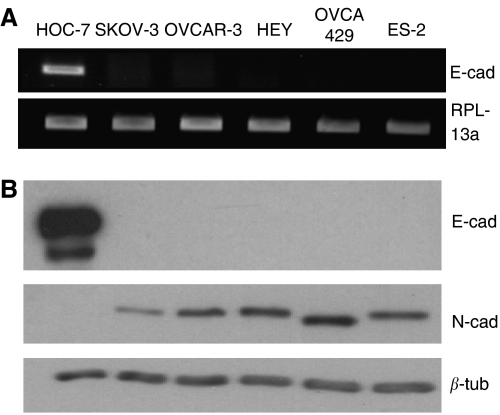
Expression of E- and N-cadherin by ovarian cancer cell lines. (**A**) RT–PCR for E-cadherin. RPL13a amplification verified equal amounts of cDNA template. (**B**) Western blot analysis for E- and N-cadherin. Blots were reprobed with *β*-tubulin to verify equal loading.

Both RT–PCR and Western blot analysis of cell lysates revealed that E-cadherin expression was restricted to HOC-7 cells, whereas the other five cell lines expressed N-cadherin (Figure 5A). These findings demonstrate that E-cadherin expression is not a factor contributing to the inability of the SKOV-3 and OVCAR-3 cell lines to invade and migrate on collagen. However, E-cadherin expression may explain the lower motility and invasiveness of the MT1-expressing HOC-7 cells relative to the other invasive MT1-expressing cells.

## DISCUSSION

Our results establish a critical role for MT1 in ovarian cancer cell invasion and are the first to reveal its expression in a panel of cancer cell lines exhibiting different capacities for invasion. Endogenous MT1 expression and activity were positively correlated with invasiveness in the six ovarian cancer cell lines examined. Importantly, the levels of invasion indicated by our *in vitro* assays mirrored the reported abilities of the cell lines to form peritoneal tumours *in vivo* (Shaw *et al*, 2004). HEY, OVCA429, and ES-2 cell lines expressed the highest levels of MT1, and were consistently shown to be the most invasive in the *in vitro* assays used. These three cell lines also reproducibly formed invasive intraperitoneal tumours in mice (Shaw *et al*, 2004). Conversely, we found SKOV-3 and OVCAR-3 cells did not express MT1 and were incapable of invading polymerised collagen I matrices *in vitro*. Consistent with these results, SKOV-3 and OVCAR-3 seldom formed intraperitoneal tumours (Shaw *et al*, 2004). HOC-7 cells, which express moderate levels of MT1 (but have not been assessed for tumour-forming ability *in vivo)*, did not invade efficiently in our assays, despite having a moderate capacity to degrade the collagen I plugs within transwells.

Collagen I was selected for use in these studies as it is a principal component of the peritoneal stromal matrix, to which invading cancer cells adhere *in vivo* (Moser *et al*, 1996; Ghosh *et al*, 2002; Mochizuki *et al*, 2004). Further, matrices composed of collagen I stimulated the most rapid/extensive dissemination of ovarian cancer cells from spheroids (Burleson *et al*, 2004). Although Matrigel™ has been commonly used for invasion assays, its suitability for this purpose has recently been challenged (Hotary *et al*, 2006). This EHS sarcoma extract contains basement membrane components that reconstitute into a loose matrix stabilised predominantly by non-covalent interactions, but in critical contrast to native basement membranes, MMP activity is not required for cell penetration (Even-Ram and Yamada, 2005; Hotary *et al*, 2006). We have verified that ovarian cancer cell penetration of this synthetic matrix is independent of MMPs as evidenced by a failure of GM6001 to block cell penetration (data not shown). For this reason, Matrigel™ matrices were not utilised in this study.

We have established MT1 to be the prime determinant of ovarian cancer cell invasion both by inhibiting MT1 activity in invasive cells, and by expressing it in the non-invasive cell lines that lack its expression. As MT1-specific inhibitors have yet to be developed, and we were unable to downregulate MT1 with siRNA in these cell lines, we used the widely accepted approach of employing MMP inhibitors with differing target specificity to enable a subtractive deduction of MT1-specific activity (Nakamura *et al*, 2004; Nisato *et al*, 2005; Egawa *et al*, 2006). Invasion was unaffected by TIMP-1, which inhibits all soluble (non-transmembrane) MMPs without inhibiting MT1 (Hornebeck *et al*, 2005). In contrast, invasion was abolished by GM6001 and TIMP-2, both of which include MT1 in their inhibitory spectra (Will *et al*, 1996). Matrix metalloproteinase-2 is activated by MT1 in a TIMP-2 dependent manner (Strongin *et al*, 1995) and cooperates with MT1 in collagen clearance by further degrading denatured 3/4- and 1/4-cleaved collagen fragments generated by the collagenase activity of MT1 (Tam *et al*, 2002). Our results indicate that gelatinolytic activities of MMP-2 or MMP-9 are not required for collagen I invasion by ovarian cancer cells as treatment with the MMP-2/MMP-9 inhibitor SB-3CT caused only a small reduction in invasion. Thus, the critical role of MT1 does not involve its activation of pro-MMP-2. Rather, our findings suggest the direct cleavage of collagen I by MT1 as the critical activity necessary for invasion of this matrix. A similar dependence on MT1 and not MMP-2 has been recently reported for endothelial cell collagen I invasion (Nisato *et al*, 2005) and for *in vitro* invasion of an authentic basement membrane matrix (Hotary *et al*, 2006).

Further evidence that MT1 has a pivotal role in collagen invasion is provided by our MT1 transfection studies. Ectopic MT1 expression permitted the non-MT1-expressing SKOV-3 and OVCAR-3 cell lines to invade collagen I and markedly enhanced the invasive abilities of OVCA429 and ES-2 cells, which had moderate endogenous MT1 expression. This effect of MT1 required the presence of the active catalytic domain.

Clustering of the collagen binding integrins has been implicated in the cell surface localisation, and thus activity of MT1. The collagen I-stimulated translocation of MT1 to the cell membrane has been suggested as a rate-limiting event for pro-MMP-2 activation and invasion by DOV-13 cells (Ellerbroek *et al*, 2001). Interestingly, MT1 activity levels in our cells, as determined by pro-MMP-2 cleavage, and collagen I degradation generally reflected the constitutive levels of MT1 mRNA and protein, suggesting that potential variations in events required for MT1 surface translocation (activation) were not a major influencing factor among the cell lines examined.

It is important to note that other groups have reported endogenous MT1 expression in SKOV-3 and OVCAR-3 cells as determined by Western blot analysis. We have often observed numerous nonspecific bands migrating near the MT1 band with different commercial antibodies, including some lots of Ab815, in extracts from breast and ovarian cancer cells. For this reason, we validated MT1 expression by real-time RT–PCR using RNA extracts derived on three separate occasions, and by gelatin zymography to show pro-MMP-2 activation consistent with MT1 expression. Moreover, recent proteomic studies with these cell lines support our present results (manuscript in preparation). It is possible that differences in MT-1 expression may reflect changes in cell characteristics emerging over time in different laboratories; however, we feel it is best to support results from Western blot analysis for MT1 by additional means. Nevertheless, our studies clearly show the causal relationship between MT1 expression and invasive behaviour within the panel of six ovarian cancer cell lines used in this study.

Our initial observation that the MT1-expressing cell lines were overall more motile on a non-polymerised collagen I matrix raised the possibility that MT1 could contribute to invasion by enhancing motility as well as collagen degradation. Several studies have reported motility to be somewhat enhanced by overexpression of wild-type MT1 (Gingras *et al*, 2001; Rozanov *et al*, 2001; Cao *et al*, 2004; Takino *et al*, 2004). MT1 effects on cell motility may be exerted through integrin activation, as MT1-mediated cleavage of the vitronectin receptor *α*v*β*3 enhanced breast cancer cell motility on this substrate (Deryugina *et al*, 2002). Although *α*v*β*3 integrin is a receptor for fibronectin rather than collagen, it has been reported to be essential for endothelial cell invasion of a collagen I matrix; its participation perhaps relates to a coating of the collagen I with fibronectin or vitronectin from the serum-containing culture media or from the cells themselves, and may play a general role in cell motility on collagen I matrices (Nisato *et al*, 2005). The mechanism through which MT1 overexpression stimulated the enhanced cell motility is unclear, as controversial reports exist regarding the requirement of its catalytic activity (Gingras *et al*, 2001; Rozanov *et al*, 2001; Takino *et al*, 2004; cf. Cao *et al*, 2004). Further, an inverse relationship between matrix clearance and MT1-mediated integrin activation create a complex dual effect on motility (Deryugina *et al*, 2003). Importantly, all studies that have reported MT1 overexpression to enhance motility were conducted in cell types other than ovarian cancer cells. Our MT1 overexpression and GM6001 studies indicate that MT1 expression does not enhance ovarian cancer cell motility on collagen I and are consistent with the report that GM6001 had no effect on the motility of DOV13 ovarian cancer cells on this matrix (Ellerbroek *et al*, 2001). Thus, although the cell lines that express endogenous MT1 are more motile than those that lack MT1 expression, this relationship is correlative and not causal; MT1 does not appear to contribute to their superior motility. Rather, MT1 is likely regulated in parallel with the pathways that promote a motile phenotype.

Although cell motility was not altered by manipulation of MT1 expression or activity, migratory capacity is an important factor in determining the extent of invasion. The importance of migratory capacity in ovarian cancer metastasis is perhaps best illustrated by the fact that therapeutic strategies aimed at preventing tumour cell motility reduce peritoneal metastasis of ovarian cancer cells (Hashimoto *et al*, 2005). Motility and invasion of numerous cancer cell types is prevented by expression of the tumour-suppressor E-cadherin. Through its sequestration of the transcriptional co-activator *β*-catenin, E-cadherin inhibits Tcf/LEF transcriptional activation of numerous targets that contribute to a motile, invasive phenotype, including MT1 (Ara *et al*, 2000; Takahashi *et al*, 2002; Nawrocki-Raby *et al*, 2003a, 2003b; Hazan *et al*, 2004; Hlubek *et al*, 2004; Munshi and Stack, 2006).

The pattern of E-cadherin expression in ovarian cancer appears to be distinct from that of most other epithelial cancers. Primary ovarian carcinomas have upregulated E-cadherin and are more epithelial in character than the N-cadherin-expressing ovarian surface epithelium (mesothelium) from which they are presumed to arise. Subsequently, E-cadherin expression decreases and correlates with progression to metastatic disease, in a manner similar to other carcinomas (Auersperg *et al*, 2002; Yuecheng *et al*, 2006) indicating ovarian cancer metastasis is likely promoted by the de-differentiation that accompanies E-cadherin downregulation. It has been suggested that the inability of the SKOV-3 and OVCAR-3 ovarian cancer cell lines to invade collagen I may be due to their E-cadherin expression (Kokenyesi *et al*, 2003). However, our studies clearly show that of the cells used in the present study, only HOC-7 expressed E-cadherin, whereas the other cell lines expressed N-cadherin. The idea proposed by Kokenyesi *et al*, (2003) is consistent with the behaviour of the HOC-7 cells, which have an epithelial morphology, grow in tight clusters, and migrate as a sheet in wound-healing assays (K Sodek, unpublished observations), indicative of the strong cell–cell adhesion endowed by their E-cadherin expression. Migration of cells through a collagen I gel, as in the Kokenyesi study, or through the narrow 8 *μ*m diameter transwell pores, as used in the current study, would require cell–cell detachment, and would thus likely be impeded by E-cadherin expression.

Expression of MT1 by cancer cells in ovarian tumours has been associated with poor patient survival (Davidson *et al*, 1999; Sakata *et al*, 2000; Davidson *et al*, 2002). As with other cancers, MMP inhibitors have yielded disappointing results in ovarian cancer clinical trials, likely because they have been administered at late stages of disease and are broad-range, inhibiting MMPs that have anti-tumorigenic/anti-angiogenic activities (Overall and Lopez-Otin, 2002). Alternatively, it has been recently reported that the inhibitor concentrations required to inhibit MMP catalytic activity are far above the physiological levels achieved (Hotary *et al*, 2006). Furthermore, the MMP inhibitors marimastat and tanomastat, which have been administered in ovarian cancer trials, are selective for MMPs other than MT1 (Hidalgo and Eckhardt, 2001). The development of specific inhibitors of MT1 has proven a difficult task, as its structure is similar to other MMPs yet, more restrictive, thus inhibitors that bind MT1 also tend to bind other MMPs, especially the gelatinases (Yamamoto *et al*, 1998). However, the further development of a new class of peptidomimetic mercaptosulphide MMP inhibitors that have high selectivity for MT1 and the gelatinases holds promise for design of a specific MT1 inhibitor (Hurst *et al*, 2005; Fisher and Mobashery, 2006). An additional attractive approach is to establish and target the pathways that govern MT1 expression.

As cell motility is clearly related to the extent of invasion of ovarian cancer cells, it is important to determine factors synchronising this aspect of invasive behaviour with matrix degradation. The combination of gene expression profiling, proteomics and bioinformatical approaches as applied to these cell lines are necessary to better understand potential pathways through which MT1 expression and cell motility are regulated, and are likely to reveal new therapeutic targets for ovarian cancer.

## References

[bib1] Ara T, Deyama Y, Yoshimura Y, Higashino F, Shindoh M, Matsumoto A, Fukuda H (2000) Membrane type 1-matrix metalloproteinase expression is regulated by E-cadherin through the suppression of mitogen-activated protein kinase cascade. Cancer Lett 157: 115–121 10936671 10.1016/s0304-3835(00)00494-8

[bib2] Artym VV, Zhang Y, Seillier-Moiseiwitsch F, Yamada KM, Mueller SC (2006) Dynamic interactions of cortactin and membrane type 1 matrix metalloproteinase at invadopodia: defining the stages of invadopodia formation and function. Cancer Res 66: 3034–3043 16540652 10.1158/0008-5472.CAN-05-2177

[bib3] Auersperg N, Ota T, Mitchell GW (2002) Early events in ovarian epithelial carcinogenesis: progress and problems in experimental approaches. Int J Gynecol Can 12: 691–703 10.1046/j.1525-1438.2002.01152.x 12445245

[bib4] Aznavoorian S, Moore BA, Alexander-Lister LD, Hallit SL, Windsor LJ, Engler JA (2001) Membrane type I-matrix metalloproteinase-mediated degradation of type I collagen by oral squamous cell carcinoma cells. Cancer Res 61: 6264–6275 11507081

[bib5] Burleson KM, Hansen LK, Skubitz AP (2004) Ovarian carcinoma spheroids disaggregate on type I collagen and invade live human mesothelial cell monolayers. Clin Exp Metastasis 21: 685–697 16035613 10.1007/s10585-004-5768-5

[bib6] Cao J, Chiarelli C, Kozarekar P, Adler HL (2005) Membrane type 1-matrix metalloproteinase promotes human prostate cancer invasion and metastasis. Thromb Haemost 93: 770–778 15841326 10.1160/TH04-08-0555

[bib7] Cao J, Kozarekar P, Pavlaki M, Chiarelli C, Bahou WF, Zucker S (2004) Distinct roles for the catalytic and hemopexin domains of membrane type 1-matrix metalloproteinase in substrate degradation and cell migration. J Biol Chem 279: 14129–14139 14729674 10.1074/jbc.M312120200

[bib8] Chen WT, Wang JY (1999) Specialized surface protrusions of invasive cells, invadopodia and lamellipodia, have differential MT1-MMP, MMP-2, and TIMP-2 localization. Ann N Y Acad Sci 878: 361–371 10415741 10.1111/j.1749-6632.1999.tb07695.x

[bib9] Coussens LM, Fingleton B, Matrisian LM (2002) Matrix metalloproteinase inhibitors and cancer: trials and tribulations. Science 295: 2387–2392 11923519 10.1126/science.1067100

[bib10] Davidson B, Goldberg I, Gotlieb WH, Kopolovic J, Ben-Baruch G, Nesland JM, Berner A, Bryne M, Reich R (1999) High levels of MMP-2, MMP-9, MT1-MMP and TIMP-2 mRNA correlate with poor survival in ovarian carcinoma. Clin Exp Metastasis 17: 799–808 11089877 10.1023/a:1006723011835

[bib11] Davidson B, Goldberg I, Gotlieb WH, Kopolovic J, Ben-Baruch G, Nesland JM, Reich R (2002) The prognostic value of metalloproteinases and angiogenic factors in ovarian carcinoma. Mol Cell Endocrinol 187: 39–45 11988310 10.1016/s0303-7207(01)00709-2

[bib12] Derycke LD, Bracke ME (2004) N-cadherin in the spotlight of cell–cell adhesion, differentiation, embryogenesis, invasion and signalling. Int J Dev Biol 48: 463–476 15349821 10.1387/ijdb.041793ld

[bib13] Deryugina EI, Ratnikov BI, Strongin AY (2003) Prinomastat, a hydroxamate inhibitor of matrix metalloproteinases, has a complex effect on migration of breast carcinoma cells. Int J Cancer 104: 533–541 12594807 10.1002/ijc.10977

[bib14] Deryugina EI, Ratnikov BI, Postnova TI, Rozanov DV, Strongin AY (2002) Processing of integrin alpha(v) subunit by membrane type 1 matrix metalloproteinase stimulates migration of breast carcinoma cells on vitronectin and enhances tyrosine phosphorylation of focal adhesion kinase. J Biol Chem 277: 9749–9756 11724803 10.1074/jbc.M110269200

[bib15] Drew AF, Blick TJ, Lafleur MA, Tim EL, Robbie MJ, Rice GE, Quinn MA, Thompson EW (2004) Correlation of tumor- and stromal-derived MT1-MMP expression with progression of human ovarian tumors in SCID mice. Gynecol Oncol 95: 437–448 15581944 10.1016/j.ygyno.2004.08.032

[bib16] Egawa N, Koshikawa N, Tomari T, Nabeshima K, Isobe T, Seiki M (2006) Membrane type 1 matrix metalloproteinase (MT1-MMP/MMP-14) cleaves and releases a 22-kDa extracellular matrix metalloproteinase inducer (EMMPRIN) fragment from tumor cells. J Biol Chem 281: 37576–37585 17050542 10.1074/jbc.M606993200

[bib17] Egeblad M, Werb Z (2002) New functions for the matrix metalloproteinases in cancer progression. Nat Rev Cancer 2: 161–174 11990853 10.1038/nrc745

[bib18] Ellerbroek SM, Fishman DA, Kearns AS, Bafetti LM, Stack MS (1999) Ovarian carcinoma regulation of matrix metalloproteinase-2 and membrane type 1 matrix metalloproteinase through beta1 integrin. Cancer Res 59: 1635–1641 10197640

[bib19] Ellerbroek SM, Wu YI, Overall CM, Stack MS (2001) Functional interplay between type I collagen and cell surface matrix metalloproteinase activity. J Biol Chem 276: 24833–24842 11331272 10.1074/jbc.M005631200

[bib20] Endo K, Takino T, Miyamori H, Kinsen H, Yoshizaki T, Furukawa M, Sato H (2003) Cleavage of syndecan-1 by membrane type matrix metalloproteinase-1 stimulates cell migration. J Biol Chem 278: 40764–40770 12904296 10.1074/jbc.M306736200

[bib21] Even-Ram S, Yamada KM (2005) Cell migration in 3D matrix. Curr Opin Cell Biol 17: 524–532 16112853 10.1016/j.ceb.2005.08.015

[bib22] Fisher JF, Mobashery S (2006) Recent advances in MMP inhibitor design. Cancer Met Rev 25: 115–136 10.1007/s10555-006-7894-9 16680577

[bib23] Ghosh S, Wu Y, Stack MS (2002) Ovarian cancer-associated proteinases. Cancer Treat Res 107: 331–351 11775460 10.1007/978-1-4757-3587-1_16

[bib24] Giannelli G, Antonaci S (2000) Biological and clinical relevance of Laminin-5 in cancer. Clin Exp Metastasis 18: 439–443 11592300 10.1023/a:1011879900554

[bib25] Gingras D, Bousquet-Gagnon N, Langlois S, Lachambre MP, Annabi B, Beliveau R (2001) Activation of the extracellular signal-regulated protein kinase (ERK) cascade by membrane-type-1 matrix metalloproteinase (MT1-MMP). FEBS Lett 507: 231–236 11684104 10.1016/s0014-5793(01)02985-4

[bib26] Hashimoto K, Morishige K, Sawada K, Tahara M, Kawagishi R, Ikebuchi Y, Sakata M, Tasaka K, Murata Y (2005) Alendronate inhibits intraperitoneal dissemination in *in vivo* ovarian cancer model. Cancer Res 65: 540–545 15695397

[bib27] Hazan RB, Qiao R, Keren R, Badano I, Suyama K (2004) Cadherin switch in tumor progression. Ann N Y Acad Sci 1014: 155–163 15153430 10.1196/annals.1294.016

[bib28] Hidalgo M, Eckhardt SG (2001) Development of matrix metalloproteinase inhibitors in cancer therapy. J Natl Can Inst 93: 178–193 10.1093/jnci/93.3.178 11158186

[bib29] Hirte H, Vergote IB, Jeffrey JR, Grimshaw RN, Coppieters S, Schwartz B, Tu D, Sadura A, Brundage M, Seymour L (2006) A phase III randomized trial of BAY 12–9566 (tanomastat) as maintenance therapy in patients with advanced ovarian cancer responsive to primary surgery and paclitaxel/platinum containing chemotherapy: a National Cancer Institute of Canada Clinical Trials Group Study. Gynecol Oncol 102: 300–308 16442153 10.1016/j.ygyno.2005.12.020

[bib30] Hlubek F, Spaderna S, Jung A, Kirchner T, Brabletz T (2004) Beta-catenin activates a coordinated expression of the proinvasive factors laminin-5 gamma2 chain and MT1-MMP in colorectal carcinomas. Int J Cancer 108: 321–326 14639622 10.1002/ijc.11522

[bib31] Hornebeck W, Maquart FX (2003) Proteolyzed matrix as a template for the regulation of tumor progression. Biomed Pharmacother 57: 223–230 12888258 10.1016/s0753-3322(03)00049-0

[bib32] Hornebeck W, Lambert E, Petitfrere E, Bernard P (2005) Beneficial and detrimental influences of tissue inhibitor of metalloproteinase-1 (TIMP-1) in tumor progression. Biochimie 87: 377–383 15781325 10.1016/j.biochi.2004.09.022

[bib33] Hotary K, Li XY, Allen E, Stevens SL, Weiss SJ (2006) A cancer cell metalloprotease triad regulates the basement membrane transmigration program. Genes Dev 20: 2673–2686 16983145 10.1101/gad.1451806 PMC1578694

[bib34] Hurst DR, Schwartz MA, Jin Y, Ghaffari MA, Kozarekar P, Cao J, Sang QX (2005) Inhibition of enzyme activity of and cell-mediated substrate cleavage by membrane type 1 matrix metalloproteinase by newly developed mercaptosulphide inhibitors. Biochem J 392: 527–536 16026329 10.1042/BJ20050545 PMC1316292

[bib35] Kajita M, Itoh Y, Chiba T, Mori H, Okada A, Kinoh H, Seiki M (2001) Membrane-type 1 matrix metalloproteinase cleaves CD44 and promotes cell migration. J Cell Biol 153: 893–904 11381077 10.1083/jcb.153.5.893 PMC2174329

[bib36] Kleifeld O, Kotra LP, Gervasi DC, Brown S, Bernardo MM, Fridman R, Mobashery S, Sagi I (2001) X-ray absorption studies of human matrix metalloproteinase-2 (MMP-2) bound to a highly selective mechanism-based inhibitor. Comparison with the latent and active forms of the enzyme. J Biol Chem 276: 17125–17131 11278946 10.1074/jbc.M011604200

[bib37] Kokenyesi R, Murray KP, Benshushan A, Huntley ED, Kao MS (2003) Invasion of interstitial matrix by a novel cell line from primary peritoneal carcinosarcoma, and by established ovarian carcinoma cell lines: role of cell-matrix adhesion molecules, proteinases, and E-cadherin expression. Gynecol Oncol 89: 60–72 12694655 10.1016/s0090-8258(02)00152-x

[bib38] Koshikawa N, Minegishi T, Sharabi A, Quaranta V, Seiki M (2005) Membrane-type matrix metalloproteinase-1 (MT1-MMP) is a processing enzyme for human laminin gamma 2 chain. J Biol Chem 280: 88–93 15525652 10.1074/jbc.M411824200

[bib39] Landis SH, Murray T, Bolden S, Wingo PA (1999) Cancer statistics, 1999. [see comment] CA Cancer J Clin 49: 8–31 10200775 10.3322/canjclin.49.1.8

[bib40] Mochizuki Y, Nakanishi H, Kodera Y, Ito S, Yamamura Y, Kato T, Hibi K, Akiyama S, Nakao A, Tatematsu M (2004) TNF-alpha promotes progression of peritoneal metastasis as demonstrated using a green fluorescence protein (GFP)-tagged human gastric cancer cell line. Clin Exp Met 21: 39–47 10.1023/b:clin.0000017181.01474.35 15065601

[bib41] Mogal A AS (2006) Effects of histone deacetylase inhibitor (HDACi); trichostatin-A (TSA) on the expression of housekeeping genes. Mol Cell Probes 20: 81–86 16326072 10.1016/j.mcp.2005.09.008

[bib42] Mori H, Tomari T, Koshikawa N, Kajita M, Itoh Y, Sato H, Tojo H, Yana I, Seiki M (2002) CD44 directs membrane-type 1 matrix metalloproteinase to lamellipodia by associating with its hemopexin-like domain. EMBO J 21: 3949–3959 12145196 10.1093/emboj/cdf411 PMC126155

[bib43] Moser TL, Pizzo SV, Bafetti LM, Fishman DA, Stack MS (1996) Evidence for preferential adhesion of ovarian epithelial carcinoma cells to type I collagen mediated by the alpha2beta1 integrin. Int J Cancer 67: 695–701 8782661 10.1002/(SICI)1097-0215(19960904)67:5<695::AID-IJC18>3.0.CO;2-4

[bib44] Munshi HG, Stack MS (2006) Reciprocal interactions between adhesion receptor signaling and MMP regulation. Cancer Met Rev 25: 45–56 10.1007/s10555-006-7888-7 16680571

[bib45] Nakamura H, Suenaga N, Taniwaki K, Matsuki H, Yonezawa K, Fujii M, Okada Y, Seiki M (2004) Constitutive and induced CD44 shedding by ADAM-like proteases and membrane-type 1 matrix metalloproteinase. Cancer Res 64: 876–882 14871815 10.1158/0008-5472.can-03-3502

[bib46] Nawrocki-Raby B, Gilles C, Polette M, Bruyneel E, Laronze JY, Bonnet N, Foidart JM, Mareel M, Birembaut P (2003a) Upregulation of MMPs by soluble E-cadherin in human lung tumor cells. Int J Cancer 105: 790–795 12767064 10.1002/ijc.11168

[bib47] Nawrocki-Raby B, Gilles C, Polette M, Martinella-Catusse C, Bonnet N, Puchelle E, Foidart JM, Van Roy F, Birembaut P (2003b) E-Cadherin mediates MMP down-regulation in highly invasive bronchial tumor cells. Am J Pathol 163: 653–661 12875984 10.1016/S0002-9440(10)63692-9 PMC1868220

[bib48] Nisato RE, Hosseini G, Sirrenberg C, Butler GS, Crabbe T, Docherty AJ, Wiesner M, Murphy G, Overall CM, Goodman SL, Pepper MS (2005) Dissecting the role of matrix metalloproteinases (MMP) and integrin alpha(v)beta3 in angiogenesis *in vitro*: absence of hemopexin C domain bioactivity, but membrane-Type 1-MMP and alpha(v)beta3 are critical. Cancer Res 65: 9377–9387 16230401 10.1158/0008-5472.CAN-05-1512

[bib49] Ohuchi E, Imai K, Fujii Y, Sato H, Seiki M, Okada Y (1997) Membrane type 1 matrix metalloproteinase digests interstitial collagens and other extracellular matrix macromolecules. J Biol Chem 272: 2446–2451 8999957 10.1074/jbc.272.4.2446

[bib50] Overall CM, Lopez-Otin C (2002) Strategies for MMP inhibition in cancer: innovations for the post-trial era. Nat Rev Cancer 2: 657–672 12209155 10.1038/nrc884

[bib51] Rozanov DV, Deryugina EI, Ratnikov BI, Monosov EZ, Marchenko GN, Quigley JP, Strongin AY (2001) Mutation analysis of membrane type-1 matrix metalloproteinase (MT1-MMP). The role of the cytoplasmic tail Cys(574), the active site Glu(240), and furin cleavage motifs in oligomerization, processing, and self-proteolysis of MT1-MMP expressed in breast carcinoma cells. J Biol Chem 276: 25705–25714 11335709 10.1074/jbc.M007921200

[bib52] Sabeh F, Ota I, Holmbeck K, Birkedal-Hansen H, Soloway P, Balbin M, Lopez-Otin C, Shapiro S, Inada M, Krane S, Allen E, Chung D, Weiss SJ (2004) Tumor cell traffic through the extracellular matrix is controlled by the membrane-anchored collagenase MT1-MMP. J Cell Biol 167: 769–781 15557125 10.1083/jcb.200408028 PMC2172570

[bib53] Sakata K, Shigemasa K, Nagai N, Ohama K (2000) Expression of matrix metalloproteinases (MMP-2, MMP-9, MT1-MMP) and their inhibitors (TIMP-1, TIMP-2) in common epithelial tumors of the ovary. Int J Oncol 17: 673–681 10995877

[bib54] Seiki M (2003) Membrane-type 1 matrix metalloproteinase: a key enzyme for tumor invasion. Cancer Lett 194: 1–11 12706853 10.1016/s0304-3835(02)00699-7

[bib55] Shaw TJ, Senterman MK, Dawson K, Crane CA, Vanderhyden BC (2004) Characterization of intraperitoneal, orthotopic, and metastatic xenograft models of human ovarian cancer. Mol Ther 10: 1032–1042 15564135 10.1016/j.ymthe.2004.08.013

[bib56] Sivridis E, Giatromanolaki A, Koukourakis MI (2004) "Stromatogenesis" and tumor progression. Int J Surg Pathol 12: 1–9 14765266 10.1177/106689690401200101

[bib57] Sounni NE, Noel A (2005) Membrane type-matrix metalloproteinases and tumor progression. Biochimie 87: 329–342 15781320 10.1016/j.biochi.2004.07.012

[bib58] Stack MS, Ellerbroek SM, Fishman DA (1998) The role of proteolytic enzymes in the pathology of epithelial ovarian carcinoma. Int J Oncol 12: 569–576 9472094 10.3892/ijo.12.3.569

[bib59] Strongin AY, Collier I, Bannikov G, Marmer BL, Grant GA, Goldberg GI (1995) Mechanism of cell surface activation of 72-kDa type IV collagenase. Isolation of the activated form of the membrane metalloprotease. J Biol Chem 270: 5331–5338 7890645 10.1074/jbc.270.10.5331

[bib60] Takahashi M, Tsunoda T, Seiki M, Nakamura Y, Furukawa Y (2002) Identification of membrane-type matrix metalloproteinase-1 as a target of the beta-catenin/Tcf4 complex in human colorectal cancers. Oncogene 21: 5861–5867 12185585 10.1038/sj.onc.1205755

[bib61] Takino T, Miyamori H, Watanabe Y, Yoshioka K, Seiki M, Sato H (2004) Membrane type 1 matrix metalloproteinase regulates collagen-dependent mitogen-activated protein/extracellular signal-related kinase activation and cell migration. Cancer Res 64: 1044–1049 14871836 10.1158/0008-5472.can-03-1843

[bib62] Tam EM, Wu YI, Butler GS, Stack MS, Overall CM (2002) Collagen binding properties of the membrane type-1 matrix metalloproteinase (MT1-MMP) hemopexin C domain. The ectodomain of the 44-kDa autocatalytic product of MT1-MMP inhibits cell invasion by disrupting native type I collagen cleavage. J Biol Chem 277: 39005–39014 12145314 10.1074/jbc.M206874200

[bib63] Ueda J, Kajita M, Suenaga N, Fujii K, Seiki M (2003) Sequence-specific silencing of MT1-MMP expression suppresses tumor cell migration and invasion: importance of MT1-MMP as a therapeutic target for invasive tumors. Oncogene 22: 8716–8722 14647466 10.1038/sj.onc.1206962

[bib64] Will H, Atkinson SJ, Butler GS, Smith B, Murphy G (1996) The soluble catalytic domain of membrane type 1 matrix metalloproteinase cleaves the propeptide of progelatinase A and initiates autoproteolytic activation^*^ Regulation by TIMP-2 and TIMP-3. J Biol Chem 271: 17119–17123 8663332 10.1074/jbc.271.29.17119

[bib65] Wu YI, Munshi HG, Sen R, Snipas SJ, Salvesen GS, Fridman R, Stack MS (2004) Glycosylation broadens the substrate profile of membrane type 1 matrix metalloproteinase. J Biol Chem 279: 8278–8289 14670950 10.1074/jbc.M311870200

[bib66] Yamamoto M, Tsujishita H, Hori N, Ohishi Y, Inoue S, Ikeda S, Okada Y (1998) Inhibition of membrane-type 1 matrix metalloproteinase by hydroxamate inhibitors: an examination of the subsite pocket. J Med Chem 41: 1209–1217 9548812 10.1021/jm970404a

[bib67] Yuecheng Y, Hongmei L, Xiaoyan X (2006) Clinical evaluation of E-cadherin expression and its regulation mechanism in epithelial ovarian cancer. Clin Exp Met 23: 65–74 10.1007/s10585-006-9020-3 16826427

[bib68] Zigrino P, Drescher C, Mauch C (2001) Collagen-induced proMMP-2 activation by MT1-MMP in human dermal fibroblasts and the possible role of alpha2beta1 integrins. Eur J Cell Biol 80: 68–77 11211937 10.1078/0171-9335-00134

